# Can Molecular Biomarkers Change the Paradigm of Pancreatic Cancer Prognosis?

**DOI:** 10.1155/2016/4873089

**Published:** 2016-09-01

**Authors:** Javier Martinez-Useros, Jesus Garcia-Foncillas

**Affiliations:** Translational Oncology Division, OncoHealth Institute, Health Research Institute, University Hospital Fundación Jiménez Díaz-UAM, Madrid, Spain

## Abstract

Pancreatic ductal adenocarcinoma is one of the most lethal types of tumour, and its incidence is rising worldwide. Although survival can be improved when these tumours are detected at an early stage, this cancer is usually asymptomatic, and the disease only becomes apparent after metastasis. The only prognostic biomarker approved by the FDA to date is carbohydrate antigen 19-9 (CA19-9); however, the specificity of this biomarker has been called into question, and diagnosis is usually based on clinical parameters. Tumour size, degree of differentiation, lymph node status, presence of distant metastasis at diagnosis, protein levels of KI-67 or C-reactive protein, and mutational status of* P53*,* KRAS*, or* BRCA2* are the most useful biomarkers in clinical practice. In addition to these, recent translational research has provided evidence of new biomarkers based on different molecules involved in endoplasmic reticulum stress, epithelial-to-mesenchymal transition, and noncoding RNA panels, especially microRNAs and long noncoding RNAs. These new prospects open new paths to tumour detection using minimally or noninvasive techniques such as liquid biopsies. To find sensitive and specific biomarkers to manage these patients constitutes a challenge for the research community and for public health policies.

## 1. Introduction

Pancreatic ductal adenocarcinoma (PDAC) is the fourth leading cause of cancer death in both sexes in the USA. In 2014, the number of deaths from PDAC in the USA was 39,590, and PDAC is the cause of 227,000 deaths per year worldwide [1, 2]. Furthermore, a statistical analysis carried out from 2001 to 2010 indicates that death rates are rising [3]. Survival can be improved when tumours are detected at an early stage: it has been reported that 5-year survival rate is 50% when tumours are <2 cm [4] and close to 100% for tumours <1 cm [5]. However, PDAC is usually asymptomatic, and the disease only becomes apparent after the tumour invades surrounding tissues or metastasises to distant organs [6].

Cigarette smoking is the leading preventable extra-genetic cause of PDAC and is believed to account for 20% of PDAC cases [7]. Smoking shows a dose-related effect on tumour development, increasing the risk of PDAC by 25% compared to nonsmokers [8]. Chronic pancreatitis also increases the risk of PDAC, causing a cumulative risk of 4% after 20 years [9]. Additionally, diabetes was recently considered a potential and early symptom of PDAC, as the disease is observed in approximately 30% of all patients [10]. Also, several studies have investigated the specific role of infectious agents that affect PDAC. Of these, the strongest association has been reported for* Helicobacter pylori*: a meta-analysis comprising seven studies found that presence of* Helicobacter pylori* was correlated with as much as a 65% increased risk of developing PDAC [11, 12].

For the moment, surgical resection remains the best option to manage PDAC, and survival can be predicted based on the pathological characteristics of the tumour such as T, N, and M stage, grade of differentiation, or positive resection margins [13]. However, there is a lack of validated postsurgical prognostic or predictive markers to be used in patient management [14]. In this context, several reports of prognostic molecular biomarkers have appeared in the literature. They include SMAD4, MUC1, and also predictive markers including SPARC, HuR, or members of the BRCA2 family [15–17]. However, new high throughput genetic profiling platforms have become a useful tool for analysing whole DNA, RNA, and other factors that may or may not be translated into protein, mainly microRNAs (miRNA). In the era of genomics, transcriptomics, and proteomics, these methodologies have helped to elucidate potential biomarkers to manage patients with PDAC.

**Figure 1:**
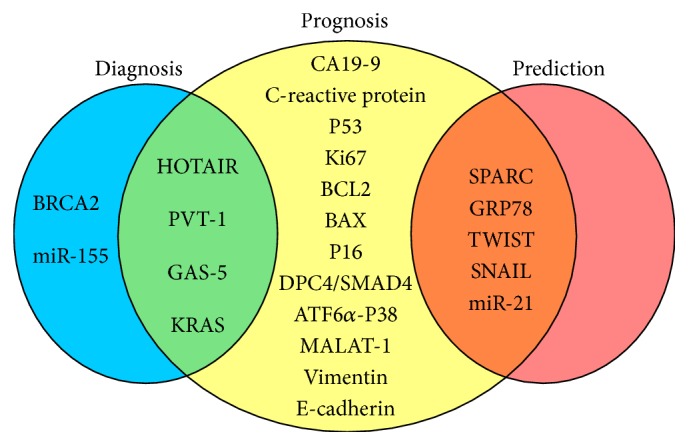
Molecular biomarkers in PDAC. Most of the molecular biomarkers may have multiple functions. Some prognosis biomarkers could be used both for diagnosis (HOTAIR, PVT-1, GAS-5, and KRAS) and for predicting treatment response (SPARC, GRP78, TWIST, SNAIL, and miR-21).

The NIH Biomarker Working Group defined biomarkers as “a characteristic i.e., objectively measured and evaluated as an indicator of normal biological processes, pathogenic processes or pharmacologic responses to a therapeutic intervention [18].” Biomarkers can be categorised as diagnostic, prognosis, or predictive based on their function; however, some biomarkers may have multiple functions (Figure 1, Table 1). Diagnostic biomarkers are able to identify early high-risk premalignant lesions. Prognostic biomarkers provide information about disease outcome in surgically resected individuals not treated with chemotherapy, radiotherapy, or their combinations. Predictive markers can discriminate between responders to a given treatment and nonresponders.

## 2. Biomarkers Based on Clinical Variables

Very few biomarkers have been introduced in the routine clinical management of PDAC. The most commonly used are based on clinical variables such as ECOG and other variables like levels of CA19-9. The way the disease affects a patient's daily living abilities is determined according to the ECOG (Eastern Cooperative Oncology Group) classification. ECOG has been considered as an important independent prognostic factor for patient outcome. This system published in 1982 was agreed upon as standard criteria to quantify functionality of cancer patients in terms of their ability to have daily regular and physical activity or provide self-care in order to determine the ability to receive a certain treatment (Table 2) [61]. Several clinical trials demonstrated that poor ECOG is an independent negative prognostic factor in PDAC. Thus, a baseline ECOG value of 2 was reported as an independent adverse prognostic factor for survival (HR = 1.735; *P* < 0.001) in one study that compared gemcitabine in combination with oxaliplatin to gemcitabine alone [19]. A phase III clinical trial comparing gemcitabine in combination with tipifarnib to gemcitabine plus placebo revealed ECOG 0 to be a better prognostic factor associated with survival (HR = 0.53; *P* < 0.001) [20]. Recently, high ECOG (HR = 2.26; *P* = 0.001) was associated with poorer overall survival in patients treated with FOLFOXIRI as a first-line treatment [21].

In addition to these, new molecular biomarkers have appeared which can dissect disease information. Deletions, mutations, translocations, amplifications, overexpression or downregulation of DNA, RNA, protein, or noncoding RNA factors are the most commonly described in scientific reports. A selection of these molecular biomarkers is summarised below to broaden the understanding of their functions and potential clinical uses.

## 3. Carbohydrate Antigen 19-9

Elevated serum levels of carbohydrate antigen 19-9 (CA19-9) have been confirmed as a prognostic biomarker for PDAC, since patients with high values for this antigen presented statistically significant poor survival. In one study, it was suggested that elevated preoperative serum levels of CA19-9 could predict time to recurrence after surgery (*P* = 0.0049) [22]. To date, the only FDA-approved biomarkers for resectable PDAC are preoperative levels of CA19-9, and this biomarker is used for both early detection and establishing prognosis (*P* = 0.003) [23]. CA19-9 shows higher sensitivity for PDAC [62] compared to carcinoembryonic antigen (CEA), carbohydrate antigen 50 (CA-50), and DUPAN-2 [63, 64]. However, the specificity of this marker has been called into question since other clinical events such as biliary obstruction can increase CA19-9 serum levels [65] and because up to 10% of the population cannot synthesise this antigen [66]. Nevertheless, CA19-9 is currently considered the best serum marker for PDAC [67].

## 4. C-Reactive Protein

C-reactive protein is a protein produced by the liver as part of the systemic inflammatory response and has been considered a useful biomarker based on detection of inflammation [68, 69]. High concentration of C-reactive protein has been previously associated with shorter survival in unresectable PDAC [24, 25]. Concerning resectable PDAC, it has been reported that C-reactive protein levels ≤10 mg/L after surgery predicted better disease outcome (*P* < 0.001). However, elevated preoperative C-reactive protein associated with higher tumour size (*P* < 0.05), vascular invasion (*P* < 0.05), and poor differentiation (*P* < 0.05) [26]. In another study, high concentration of C-reactive protein (>5 mg/L) associated with a significantly reduced survival in unresectable PDAC (*P* = 0.027) independently of biliary tract obstruction, although no association was found in resectable cohort [27]. Recently, it has been reported that the ratio between C-reactive protein and albumin is a significant prognostic biomarker for resectable PDAC after operation (*P* = 0.035), together with TNM classification (*P* = 0.003) [70]. Furthermore, C-reactive protein at low (<0.5 mg/L), medium (≥0.5 and <2.0 mg/L), and high levels (≥2.0 mg/L) is associated with good, moderate, and poor survival, respectively [28].

## 5. SPARC

The secreted protein acidic and rich in cysteine, abbreviated as SPARC, is a crucial glycoprotein for PDAC proliferation, invasion, metastasis, and chemoresistance [71, 72]. In one study, expression of SPARC was not associated with patient prognosis (*P* = 0.13), although the authors report that patients whose tumour stroma expressed SPARC had shorter median survival than patients whose tumour stroma lacked this expression (15 months versus 30 months, resp., *P* < 0.001) [29]. Another study supports the role of SPARC as a prognostic factor, with a similar median survival to that of the aforementioned study (11.5 versus 25.3 months; *P* = 0.020) [30]. Further research has associated stromal and cytoplasmic SPARC expression with short survival and poor response to gemcitabine [31].

## 6. KRAS

One of the causes of mutation in* KRAS* is the uncontrolled activation of RAS via Hedgehog pathway through SMO [73]. However, Hedgehog is not enough to trigger the RAS pathway in pancreatic malignancies [74]. Another factor that interacts and regulates the KRAS variants G12V and G12D is ribonucleoprotein HNRNPA2B1 [75]. PDAC has the highest incidence of* KRAS* mutation of all types of tumours, and more than 50% of patients could exhibit this abnormality [32, 76]; additionally, the* KRAS* mutation is considered a critical event for the initiation of this type of cancer [77].

The FDA approved Therascreen (Qiagen) and Cobas 480 (Roche) assays to detect* KRAS* mutations status [78, 79]. One study that compared both methodologies showed 98% of concordance between them, although Cobas 480 identified other mutations that were not detected by initial Therascreen assay [80]. Apart from real-time PCR-based assays [81, 82], other methodologies based on pyrosequencing [83, 84] have appeared to detect higher number of* KRAS* mutations (Table 3).

One study performed with 272 patients with resectable PDAC reported the following incidence in the different* KRAS* mutations: wild type 46.2%; GAT 31.2%; GTT 14.5%; CGT 5.6%; TGT 1.7%; CTG 0.4%; and AGT 0.4% [32]. Mutational status is an independent biomarker for PDAC at multiple steps, mainly for diagnosis and prognosis, although some mutations should be taken into consideration as predictive biomarker to specific drugs [85]. Mutation G12D indicates poor prognosis (HR = 1.44; *P* = 0.01) [33]. Recently, determination of* KRAS* was performed in circulating tumour cells (CTCs) or in plasma circulating DNA (ctDNA) to determine PDAC prognosis; the results of this study have confirmed the utility of liquid biopsy as a promising material for diagnosis [34, 35].

## 7. P53

The P53 phosphoprotein encoded by the gene* TP53* is a nuclear factor that inhibits cell proliferation through activation of apoptosis [86].* TP53* is mutated in 50% and 75% of PDAC tumour cells [87, 88]. Loss of P53 has been argued to be a negative prognostic factor in pancreatic neoplasm [89–91]. However, the relation between* TP53* mutation status and clinical outcome is rather controversial, so its role as a prognostic biomarker has yet to be validated [36–40, 92, 93]. P53 overexpression showed a marked trend toward significance when compared to survival (*P* = 0.07); however, its high hazard ratio (HR = 1.8) suggests that it may be a poor prognostic factor for PDAC [36]. Another study showed statistically significant differences in disease-free survival between patients with and without P53 overexpression (*P* = 0.029), although this comparison failed to reach clinical significance, leading the authors to conclude that they did not have enough evidence in support of P53 as a prognosis factor [40].

## 8. KI-67

Ki-67 is a protein that is not expressed in resting cells, but it is present during all cell cycle phases, including G1, S, G2, and mitosis. This fact makes it an excellent clinical marker for determining the growth fraction of a tumour [94]. Ki-67 positive expression has been associated with pathological characteristics such as TNM stage, surgical resectability, or tumour grade in pancreatic cancer [42]. Kim et al. reported a statistical association between Ki-67 expression and recurrence after surgery within 1 year (*P* = 0.029) [43]. In contrast, this marker, when quantified by immunohistochemistry [37, 44] or by flow cytometry [45], seems not to have any association with survival.

## 9. BCL2 and BAX

BCL2 acts as an inhibitor of mitochondrial apoptosis, while BAX has been reported as a proapoptotic factor. Preclinical studies have concluded that increased BCL2 expression correlated with apoptotic resistance and malignant phenotype in pancreatic cancer [95, 96]. Interestingly, as opposed to other neoplasms, BCL2 in PDAC has been clinically associated with better outcome and longer survival [38, 41]. Contrary to these results, another report did not show correlation between BCL2 and survival improvement [46]. On the other hand, the same study suggested BAX expression as a strong indicator of longer survival (*P* < 0.001) even when BAX and BCL2 were found to be overexpressed in pancreatic tumour cells [46]. Thus, it seems that the role of BCL2 in pancreatic cancer progression is still unclear, and further research is needed.

## 10. P16

Encoded by the* CDKN2A* gene, P16 is a tumour suppressor factor that plays a crucial role in cell cycle regulation [97, 98]. Preclinical studies on pancreatic cancer cell lines and xenografts have reported several alterations concerning P16 that include homozygous deletions, point mutations, and inactivation by P16 promoter methylation [92]; most such alterations are accompanied by loss of the wild-type allele [99]. Clinical studies with PDAC patients support that lack of P16 protein expression is associated with advanced disease stage and poor survival (*P* < 0.05) [39, 47]. Furthermore, P16 expression may differentiate chronic pancreatitis from PDAC that frequently lacks P16 [48]. Interestingly, Ohtsubo et al. found P16 positivity in 77% of PDAC tumours. However, they settled on an association between* P16* mutation or hypermethylation and shorter patient survival (*P* < 0.05) [49]. Concerning associations with clinicopathological characteristics, studies have shown several discrepancies. Loss of P16 expression was associated with lymph node metastasis (*P* = 0.040), more advanced stage (*P* = 0.015) [40], and greater tumour size [49]. On the other hand, loss of P16 is associated with poor differentiation grade (*P* < 0.01) but not with other clinicopathological characteristics, including clinical stage, tumour location, resectability, and survival [48].

## 11. DPC4/SMAD4

DPC4 (Deleted in Pancreatic Cancer 4) is a truncated protein encoded by a mutated form of the* SMAD4* gene located in the human chromosome 18. It has been considered a tumour suppressor gene and has been found to be highly mutated in colorectal cancer and PDAC [100]. The signalling pathway triggered by TGF-*β* has become of great interest concerning DPC4. A nonsense mutation in* SMAD4* generates a C-terminal truncation of 38 amino acids in the DPC4 protein, and it has been detected in 55% of patients with PDAC [101]. The mutant DPC4 is unable to be recruited to DNA by transcription factors and thus cannot form transcriptionally active DNA-binding complexes [102]. These mutations activate the RB pathway involved in cellular proliferation [103]. It has been suggested that inactivation of* SMAD4* occurs as a late event in neoplastic progression [104]. DPC4 inactivation resulted in a reduction in survival after surgical resection in PDAC (*P* = 0.047) [32]. Tascilar et al. also confirmed that PDAC patients with SMAD4 protein expression had significantly longer survival than those lacking expression of the protein (*P* = 0.03) [50]. Furthermore, the frequency of loss of SMAD4 expression is different in various locations of the hepatobiliopancreatic cancers, so tumour origin may merit consideration analysing this factor [104]. In contrast, another study suggested that preoperative assessment of* SMAD4* mutation associated with resectability (*P* < 0.0001) and with improved survival (*P* < 0.0001) [51].

## 12. BRCA2

*BRCA2* is a tumour suppressor gene identified as a factor for heritable cancer susceptibility [105]. The role of* BRCA2* is focused on regulation of* RAD51* recombination in response to DNA damage and regulates sister chromatid cohesion and/or alignment [106]. Initially,* BRCA2* mutations were associated with breast and ovarian cancer [107, 108], but these alterations were also associated with risk of familial PDAC. Hahn et al. reported that 19% of the families they studied (range 7–39%) had either a mutation or a variant of* BRCA2* [109]. Furthermore, the probability of finding a germline mutation of* BRCA2* in a PDAC patient is between 6% and 12% when the patient has a first-degree relative diagnosed with PDAC [52]. The most common mutations found in pancreatic cancer patients are the 6174delT frameshift mutation, 6158insT mutation, splice site mutation 16-2A>G, and the splice site mutation 15-1G>A [52, 110].* BRCA2* inactivation has been reported to be a late event in pancreatic tumorigenesis [111] and suffices to initiate PDAC driven by* KRAS* mutation G12D or disrupted* TP53* [112, 113].

It seems that* BRCA2* is a high-risk factor for pancreatic cancer development but has not been related to patient outcome or treatment response.

## 13. Noncoding RNAs

Over the last few years, noncoding RNA (ncRNA), especially microRNAs (miRNAs) and long noncoding RNAs (lncRNAs), has become a new diagnostic, prognostic, and predictive tool for pancreatic cancer. Several miRNAs have been related to cell proliferation, invasion, and metastasis, the most relevant of which are miR-21, miR-155, and miR-34. The overexpression of miR-21 was associated with a shorter disease-free survival in patients who received adjuvant gemcitabine after surgical resection [114], and miR-21 overexpression predicts resistance to 5-fluorouracil [115]. Furthermore, high miR-21 levels in plasma were associated with poor outcome in those patients treated with induction chemotherapy followed by chemoradiotherapy [53]. MiR-155 was found to be overexpressed in PDAC and could be used as an early diagnostic biomarker [54]. Moreover, miR-155 represses expression of nuclear protein 1 induced by P53 (TP53INP1), and it has been shown how its restoration inhibits PDAC tumour development [116]. Pang et al. reported that miR-155 is able to reprogram normal fibroblasts into pancreatic cancer-associated fibroblasts [117]. These findings highlight the great potential of miR-155 as a future drug target. MiR-34 is able to restore partial activity of P53 in* P53*-deficient human pancreatic cancer cells [118].

HOTAIR, PVT-1, MALAT-1, and GAS5 are some of most widely studied lncRNAs in pancreatic cancer. Concerning HOTAIR, its overexpression has been described as a poor prognostic factor in PDAC and recently has been proposed as a salivary biomarker for early diagnosis with PVT-1 expression. Surprisingly, both lncRNAs were downregulated after surgical resection, which suggests their potential for use as tumour recurrence biomarkers after operation [55]. MALAT-1 is potential oncogenic lncRNA involved in proliferation, migration, and invasion [119] and promotes undifferentiated phenotype of pancreatic tumour cells [120]. GAS5 (growth arrest-specific 5) is a potential tumour suppressor factor that negatively regulates CDK6 and is significantly decreased in PDAC tissues compared to untransformed tissues [121].

All the aforementioned miRNAs and lncRNAs could serve as diagnostic and prognostic factors, complementing clinical and pathological parameters in the effort to predict the outcome of patients with pancreatic cancer. Moreover, these factors could be quantified from a whole panel and detected from biofluids, thus making them easily implemented in routine clinical diagnosis [122].

## 14. Endoplasmic Reticulum Stress Response Proteins

The main functions of endoplasmic reticulum (ER) include synthesis, folding, and modification of proteins [123]. ER stress is induced by glucose deprivation, oxidative stress, or infection. These phenomena lead to accumulation of unfolded or misfolded proteins in the ER lumen and trigger pancreatic cell dysfunction and apoptosis [124]. To counteract ER stress and induce survival, a response mechanism has emerged [125]. ATF6*α* and GRP78 are proteins that are needed to induce response to ER stress. In normal conditions, ATF6*α* is linked to GRP78; however, in conditions of ER stress, which are critical for pancreatic cells, both proteins dissociate. ATF6*α* is activated in Golgi apparatus [126], migrates to the nucleus, and transcribes survival genes to neutralise ER stress, avoiding apoptosis and promoting cell survival [127, 128]. Furthermore, ATF6*α* is considered an important component in the VEGF-induced vascularization and induces tumour cell survival and angiogenesis [129]. Recently, our group reported a protein expression signature based on high expression of ATF6*α* and low expression of P38 as a poor prognosis biomarker associated with shorter time to recurrence after surgery for resectable PDAC [56]. GRP78 is a member of the heat-shock protein 70 (HSP70) family and acts as a chaperone that promotes cell proliferation, invasion, metastasis, and drug resistance in multiples types of cancer [130]. In PDAC, GRP78 has been suggested as a poor prognosis biomarker due to its role in proliferation, migration, and invasion of tumour cells [57] and as a predictive factor for chemoresistance to gemcitabine-based treatment [131]. Such findings open possibilities for new therapeutic strategies based on blocking the activity of GRP78.

## 15. Epithelial-to-Mesenchymal Transition Factors

Epithelial-to-mesenchymal transition (EMT) involves the changes that allow conversion from epithelial-to-mesenchymal-like phenotype [132]. In pancreatic tumours, an increased number of EMT positive cells are associated with poor survival [133]. It is one of the phenomena that subserve stimulation of tumour cells to metastasise to distant organs in the early stages of disease [134, 135]. Some of the crucial factors involved in EMT are TWIST and SNAIL [135–137]. These two factors are necessary for initiation and progression of primary PDAC, their downregulation has been reported to increase survival in preclinical models, and they have also been reported to confer sensitivity to gemcitabine and irradiation [138–142]. However, knockout of these EMT factors does not reduce metastasis in PDAC [138]. On the other hand, vimentin and E-cadherin are also considered significant proteins associated with EMT. Vimentin expression in tumour cells is a sign of mesenchymal differentiation [143] and then associated with shorter survival [144]. In fact, vimentin expression in tumour cells from resected PDAC patients is an indicator of poor outcome (*P* < 0.01) and was associated with poorly differentiated tumour phenotype (*P* < 0.01) [58]. The lack of E-cadherin expression is linked to both poor differentiation tumour histology and poor outcome in PDAC patients [59, 145]. In one study, partial and complete loss of E-cadherin expression showed statistically significant association with poor survival of PDAC patients (*P* = 0.009 and *P* = 0.005, resp.) [60]. These findings suggest that some proteins involved in EMT could be considered as biomarkers of poor prognostic in PDAC and subsequently be potentially used to design target-specific drugs in the near future.

## 16. Conclusions

PDAC generally arises from other neoplasms, including pancreatic intraepithelial and intraductal papillary mucinous and mucinous cystic neoplasms [146]. An early diagnosis and the possibility of resection are the milestones for management of these aggressive neoplasms. To date, both diagnosis and prognosis are based on clinicopathologic parameters like tumour size, grade of differentiation, lymph node status, or presence of distant metastasis at diagnosis [13]. Recent advances in translational research are scarcely implemented in routine clinical practice, and only those patients with high risk for development of PDAC gain access to genetic screening [147].

After surgical resection, there are no validated prognostic or predictive markers to be used in patient management [14]. Used widely, CA19-9 is the only FDA-approved biomarker in PDAC [23]. By contrast, its low specificity brings a high number of false positives, which has caused its utility to be called into question, and its use is restricted to detection of recurrence after operation [148]. Recently, a three-marker signature based on levels of CA19-9, IGF-1, and albumin has shown a sensitivity of 93.6% and specificity of 95% when differentiating PDAC patients from other pancreatic diseases [149].

Novel molecular biomarkers must allow for quantification by means of minimally or noninvasive techniques. New molecules detected in liquid biopsies will be used to diagnose PDAC patients and will replace single markers with multimarker panels (Figure 1) [122].

Protein detection has been the gold-standard methodology for pathological diagnosis. Nowadays, immunohistochemistry is losing favour relative to qRT-PCR, and it shows that* in situ* hybridisation, microarray, and deep-sequencing will be considered the best tools for pathological diagnosis in the future (Table 1).

On the other hand, biomarkers studies sometimes lead to controversial results. Therefore, new biomarkers and larger validation cohorts are required. In addition, only biomarkers that combine high-sensitivity and specificity and being highly cost-effective will be incorporated in healthcare systems.

## Figures and Tables

**Table 1:** Published studies of PDAC biomarkers with potential value in clinical practice.

Name (biomarker type)	Molecule/method	Author	Clinical trial	*N*	Endpoint	*P* value	Reference
ECOG (prognosis)	Clinical classification/scale	Louvet et al.	Phase III	313	Gemcitabine/oxaliplatin	<0.001	[19]
		Van Cutsem et al.	Phase III	688	Gemcitabine/tipifarnib	<0.001	[20]
		Vivaldi et al.	Prospective	137	FOLFOXIRI	=0.001	[21]

CA19-9 (prognosis)	Protein/serum levels > or = 50 U/mL	Kang et al.	Retrospective	61	Survival after surgery	=0.0049	[22]
	Protein/serum levels > or = 130 U/mL	Maithel et al.	Prospective	491	Tumour unresectability	=0.005	[23]

C-reactive protein (prognosis)	Protein/serum levels > or = 5 mg/dL	Engelken et al.	Retrospective	51	Survival	=0.001	[24]
	Protein/serum levels > 10 mg/dL	Falconer et al.	Retrospective	102	Survival	=0.001	[25]
	Protein/serum levels > 10 mg/dL	Jamieson et al.	Retrospective	65	Survival	<0.001	[26]
	Protein/serum levels > 5 mg/dL	Pine et al.	Retrospective	199	Survival	=0.027	[27]
	Protein/serum levels ≥ 2.0 mg/dL	Mitsunaga et al.	Retrospective/prospective	421	Survival	<0.01	[28]

SPARC (predictor/prognosis)	Protein/IHC	Infante et al.	Retrospective	299	Survival	<0.001	[29]
		Gundewar et al.	Retrospective	88	Survival	=0.020	[30]
		Sinn et al.	Phase III	160	Survival/gemcitabine	=0.033	[31]

KRAS (diagnosis/prognosis)	DNA or ctDNA/real-time PCR	Shin et al.	Retrospective	272	Survival after surgery	=0.001	[32]
		Bournet et al.	Retrospective	219	Survival	=0.01	[33]
		Tjensvoll et al.	Prospective	14	Survival	=0.01	[34]
		Kinugasa et al.	Prospective	141	Survival	=0.002	[35]

P53 (prognosis)	Protein/IHC, DNA/real-time PCR	DiGiuseppe et al.	Retrospective	48	Survival	=0.07	[36]
		Mäkinen et al.	Retrospective	59	Survival	=n.s.	[37]
		Nio et al.	Retrospective	63	Survival	=n.s.	[38]
		Gerdes et al.	Retrospective	62	Survival	=n.s.	[39]
		Jeong et al.	Retrospective	44	Survival	=0.029	[40]
		Bold et al.	Retrospective	70	Survival	=0.01	[41]

KI-67 (prognosis)	Protein/IHC	Lundin et al.	Retrospective	133	Survival	=0.0008	[42]
		Kim et al.	Retrospective	34	1-year recurrence after surgery	=0.029	[43]
		Sagol et al.	Retrospective	45	Survival	=n.s.	[44]
		Stanton et al.	Retrospective	33	Survival	=n.s.	[45]

BCL2 (prognosis)	Protein/IHC	Nio et al.	Retrospective	63	Survival	=n.s.	[38]
		Bold et al.	Retrospective	70	Survival	=0.01	[41]
		Friess et al.	Retrospective	78	Survival	=n.s.	[46]

BAX (prognosis)	Protein/IHC	Friess et al.	Retrospective	78	Survival	<0.001	[46]

P16 (diagnosis/prognosis)	Protein/IHC	Naka et al.	Retrospective	32	Survival	<0.05	[47]
		Hu et al.	Retrospective	62	Chronic pancreatitis	<0.01	[48]
		Ohtsubo et al.	Retrospective	60	Survival	<0.05	[49]

DPC4/SMAD4 (prognosis)	Protein/IHC, DNA/real-time PCR	Shin et al.	Retrospective	272	Survival after surgery	=0.047	[32]
		Tascilar et al.	Retrospective	249	Survival after surgery	=0.03	[50]
		Biankin et al.	Retrospective	129	Tumour unresectability and survival	<0.0001	[51]

BRCA2 (diagnosis)	DNA/real-time PCR	Murphy et al.	Retrospective	31	Familial pancreatic cancer	<0.001	[52]

miRNA-21 (predictive)	RNA/real-time PCR	Khan et al.	Phase II	17	Survival/cetuximab	=0.032	[53]

miRNA-155 (diagnosis)	RNA/real-time PCR, LNA-ISH	Habbe et al.	Retrospective	79	Early diagnosis	<0.05	[54]

HOTAIR (diagnosis)	RNA/real-time PCR	Xie et al.	Retrospective	130	Liquid biopsy diagnosis	<0.001	[55]

PVT-1 (diagnosis)	RNA/real-time PCR	Xie et al.	Retrospective	130	Liquid biopsy diagnosis	<0.001	[55]

ATF6*α*-P38 (prognosis)	Protein/IHC	Martinez-Useros et al.	Retrospective	53	Recurrence after surgery	=0.008	[56]

GRP78 (prognosis)	Protein/IHC	Niu et al.	Retrospective	180	Survival	<0.05	[57]

Vimentin (prognosis)	Protein/IHC	Handra-Luca et al.	Retrospective	387	Survival after surgery	<0.01	[58]

E-cadherin (prognosis)	Protein/IHC	Li and Ji	Retrospective	59	Survival	>0.05	[59]
		Hong et al.	Retrospective	329	Survival after surgery	=0.005	[60]

*N*: number of patients; IHC: immunohistochemistry; LNA-ISH: locked nucleic acid in situ hybridization.

**Table 2:** Eastern cooperative oncology group classification of performance status.

Grade	ECOG, performance status
0	Fully active, able to carry on all predisease performance without restriction
1	Restricted in physically strenuous activity but ambulatory and able to carry out work of a light or sedentary nature, for example, light house work, office work
2	Ambulatory and capable of all self-care but unable to carry out any work activities; up and about more than 50% of waking hours
3	Capable of only limited self-care; confined to bed or chair more than 50% of waking hours
4	Completely disabled; cannot carry on any self-care; totally confined to bed or chair
5	Dead

**Table 3:** Most common methods to determine *KRAS *mutation status.

Manufacturer	Methods	*KRAS* codons	Mutations	Exons	Commercial kit	Mean sensitivity	References
Roche	Real-time PCR	12 and 13	G12A; G12D; G12R; G12C; G12S; G12V; G13D	2	Cobas® KRAS Mutation Test	2.8%	[81]
Qiagen	Pyrosequencing	12, 13, 61	G12A; G12D; G12R; G12C; G12S; G12V; G13D; Q61H; Q61L; Q61R; Q61H; Q61E	2 and 3	Therascreen® KRAS Pyro	2.3%	[83]
Qiagen	Pyrosequencing	59, 61, 117, 146	A59T; A59G; Q61H; Q61L; Q61R; Q61H; Q61E; K117N; K117N; A146T; A146P; A146V	3 and 4	RAS Extension Pyro V2	4.1%	[84]
Qiagen	Real-time PCR	12 and 13	G12A; G12D; G12R; G12C; G12S; G12V; G13D	2	THERASCREEN KRAS RGQ PCR	3.5%	[82]
